# Treatment of moderate‐to‐severe atopic eczema in adults within the U.K.: results of a national survey of dermatologists

**DOI:** 10.1111/bjd.15235

**Published:** 2017-04-16

**Authors:** K. Taylor, D.J. Swan, A. Affleck, C. Flohr, N.J. Reynolds, in collaboration with the U.K. Translational Research Network in Dermatology and the U.K. Dermatology Clinical Trials Network

**Affiliations:** ^1^ Policy, Ethics and Life Sciences Research Centre Newcastle University Newcastle upon Tyne U.K.; ^2^ Institute of Cellular Medicine Newcastle University Newcastle upon Tyne U.K.; ^3^ Newcastle University Business School Newcastle upon Tyne U.K.; ^4^ Unit for Population‐Based Dermatology Research St John's Institute of Dermatology Guy's and St Thomas' Hospital NHS Foundation Trust and King's College London U.K.; ^5^ Department of Dermatology Royal Victoria Infirmary Newcastle upon Tyne U.K.

## Abstract

**Background:**

Little is known about U.K. dermatologists' treatment approaches towards adult patients with recalcitrant moderate‐to‐severe atopic eczema.

**Objectives:**

We wanted to learn about (i) treatment approaches used for this disease in the U.K.; (ii) factors that influence treatment decisions and (iii) perceived gaps in evidence on treatment safety and efficacy, and priorities for future trials.

**Methods:**

We conducted an online survey of consultant‐level dermatologists in the U.K.

**Results:**

Sixty‐one respondents from over 30 centres reported on management of moderate‐to‐severe atopic eczema in adults, outwith the context of an acute flare. Phototherapy or psoralen–ultraviolet A was the most common therapeutic modality chosen first line (46%), and this was usually narrowband ultraviolet B. Systemic therapy was chosen as a first‐line approach by 36% of dermatologists. Azathioprine was the commonest drug reported being used as first line followed by oral corticosteroids, ciclosporin and methotrexate. Methotrexate was the most common second‐line treatment of respondents. The key factors that influenced decision making on the use of phototherapy and systemic agents were the respondent's clinical experience, results of baseline tests (systemic agents) and knowledge of both efficacy and acute and chronic side‐effect profiles. The most important evidence gaps identified were the relative effectiveness of treatments, the alternatives to current approaches and the safety of long‐term maintenance treatment. With regard to future trials, respondents suggested that priority should be given to studies involving methotrexate.

**Conclusions:**

While survey study designs have limitations, we found that phototherapy, in particular narrowband ultraviolet B, was respondents' preferred first‐line treatment for adults with recalcitrant moderate‐to‐severe atopic eczema, perhaps reflecting access to, and clinical experience of, this approach. Azathioprine is widely used as a longer‐term maintenance treatment.

## Abstract 2

**What's already known about this topic?**

- Clinical trials have shown that phototherapy is an effective treatment for moderate‐to‐severe atopic eczema in adults, but the extent to which it is used in clinical practice in the U.K. is unknown.
- Ciclosporin is currently the only licensed systemic treatment for moderate‐to‐severe atopic eczema in adults.
- It is known that other systemic drugs are widely prescribed off‐label, but the only reliable data on their use come from a systematic survey of dermatologists and paediatricians published in 2013 on the treatment of paediatric eczema across Europe.

**What does this study add?**

- The majority of U.K. consultant‐level dermatologists use phototherapy and systemic drugs for treating adults with moderate‐to‐severe atopic eczema.
- Key factors influencing the treatment decisions made by U.K. dermatologists were their own clinical experience, results of baseline tests, knowledge of treatment efficacy and knowledge of the acute and chronic side‐effect profiles of the various treatment options.
- We report the clinical trials that dermatologists would like to see performed; a study involving methotrexate was identified as a priority.

Respond to this article

Linked Comment: Drucker. *Br J Dermatol* 2017; 176:1441–1442

Atopic eczema is a disabling long‐term condition. Although it is often considered a disease of childhood, the prevalence of atopic eczema in adults is about 3–10%.^1^, ^2^ Atopic eczema is characterized by skin barrier dysfunction and chronic inflammation. The itching, associated loss of sleep and skin infections that accompany moderate‐to‐severe disease significantly impair quality of life. Although around 60% of children will go into remission by adolescence, refractory moderate‐to‐severe atopic eczema in adults usually runs a prolonged and protracted course,^3^ typically affecting over 40% of the body surface area.^4^, ^5^ While many adult patients respond to treatment with topical corticosteroids or calcineurin inhibitors and moisturizers, a significant percentage have recalcitrant disease.

Adults with moderate‐to‐severe atopic eczema refractory to topical treatments may be considered for phototherapy or systemic drug treatment. Currently, ciclosporin is the only oral drug licensed for refractory atopic eczema. It should be used only for a limited period (the British National Formulary recommends 2 months maximum) because prolonged use is associated with hypertension, renal impairment and risk of cancer.^6^, ^7^, ^8^, ^9^, ^10^, ^11^

In 2000 a systematic review^12^ highlighted the lack of therapeutic options for patients with refractory atopic eczema and underscored the need for robust testing of a range of treatments for the disease. Investigators responded with clinical trials assessing a variety of modalities of phototherapy and a number of systemic agents, often adapted from use in other autoimmune and inflammatory conditions. In 2012, the European Dermatology Forum, the European Academy of Dermatology and Venereology, the European Task Force on Atopic Dermatitis, the European Federation of Allergy, the European Society of Pediatric Dermatology and the Global Allergy and Asthma European Network published a consensus set of guidelines for the treatment of atopic eczema.^9^ This summarized the evidence base for phototherapy and systemic agents. The 2013 European TREatment of severe Atopic eczema in children Taskforce (TREAT) survey provided valuable information about treatment of atopic eczema in children across eight European countries, including the U.K.^2^ Whether or not dermatologists are following national and international guidelines on treatment of atopic eczema in adults, and indeed their degree of knowledge of the evidence base, is not known.

The aim of this study was to survey current treatment approaches by consultant‐level dermatologists for adults with refractory moderate‐to‐severe eczema across U.K. dermatology departments. Specifically we wanted to (i) learn about the hierarchy of treatment decisions made by dermatologists and the factors governing those decisions, (ii) determine the extent of use of off‐label systemic therapies and (iii) identify perceived gaps in evidence and thereby inform the design of future clinical studies.

## Patients and methods

A web‐based survey instrument was designed, based on the question set used in the paediatric TREAT study,^2^ but adapted for adult patients and including phototherapy (Appendix S1; see Supporting Information). We used commercially available survey software (SurveyMonkey) and the structure and individual questions were validated using the cognitive theory method of Willis.^13^ Each question was systematically assessed for problems with comprehension, decision and response processes, and retrieval from memory. Comprehensibility was ensured by balancing the intent of each question and the appropriateness of the use of language for the survey audience.

Assessing respondent decision processes involved ensuring clarity and avoiding formulations where respondents could give ‘socially desirable’, rather than truthful, responses. Multiple‐choice options, including ‘Don't know’, were matched to the questions and chosen to yield the quantitative information required while ensuring consistency across the survey instrument. Time periods were chosen to reflect the compromise between likely accurate recall and achieving useable data. Free‐text responses were sought to questions about future studies so that respondents were not constrained to particular views held by the authors. A piloting exercise was undertaken involving 11 dermatologists before finalizing the question set.

For the purpose of the survey, we defined moderate‐to‐severe atopic eczema as atopic eczema ‘which is not adequately controlled by standard and optimised topical treatment (including emollients, topical steroids and topical calcineurin inhibitors), apart from occasional short‐term flares caused by skin infection for example’ (Appendix S1), and all the results reported relate to this definition.

A link to the survey instrument was distributed by e‐mail to the members of both the U.K. Translational Research Network in Dermatology (U.K. TREND) and the U.K. Dermatology Clinical Trials Network (U.K. DCTN). In total, 334 e‐mails were sent, reaching the target group of U.K. consultant dermatologists and associate specialists. Some recipients were members of both networks, so in order to reduce the possibility that recipients would reply more than once, the SurveyMonkey system was configured to permit only one submission per IP address. The questionnaire was open from 3 to 27 June 2013, and 77 responses were received. Of these, two respondents were working outside of the U.K. and 14 were nonconsultant grades. As we wished to focus on U.K. practice, and responses from nonconsultant‐level grades most likely overlap with those of their supervisors, these data were excluded, leaving 61 valid responses.

To reduce potential errors resulting from inaccurate recall of patient numbers referred or treated over long time scales, respondents were offered ranges, rather than being asked to supply discrete figures. Throughout the paper we therefore provide *estimates* of the numbers of patients referred or treated. These were calculated using data provided to us by the British Association of Dermatologists (BAD) (M. Benham, personal communication), from which we conservatively estimated that there were 729 practising consultant, or equivalent grade, dermatologists in the U.K. in 2013. We used a standard statistical approach to obtain median values of group data:

$$\text{Med}=B_{i}+\left(\frac{\frac{n}{2}-{\text{cf}}_{p}}{f_{m}}\right)i$$

where B_i_ is the lower class boundary of the group containing the median, *n* is the total number of data points, cf_p_ is the cumulative frequency of the groups before the median group, f_m_ is the frequency of the median group and *i* is the group interval.

## Results

### Respondents

The 61 valid respondents comprised consultant dermatologists (74%), clinical academics (honorary consultant dermatologists; 15%) and associate specialists in dermatology (11%). Based on the membership of the U.K. DCTN and U.K. TREND, the overall response rate for this group was 18·3%. The respondents came from over 30 centres from 31 different cities across England, Wales and Scotland. The majority saw their adult dermatology patients in university teaching hospitals (56%), followed by general hospitals (41%), with the remainder seeing their patients in private practice (2%) or the community (2%).

### Number of newly referred patients and treatment choices

We asked respondents to estimate the number of newly referred adults with refractory moderate‐to‐severe atopic eczema seen over an average 3‐month period. These data are shown in Table S1 (see Supporting Information). Using these data and the formula above, we calculated the median number of new referrals of adults with moderate‐to‐severe atopic eczema per dermatologist over a 3‐month period to be 8·6. There was a trend for a slightly greater number of referrals reported by consultants from teaching compared with district hospitals, consistent with the location of dedicated atopic eczema clinics in larger centres.

### Therapy selection

All respondents reported that adult patients with refractory moderate‐to‐severe atopic eczema would be treated using either phototherapy/psoralen–ultraviolet A (PUVA) photochemotherapy or oral systemic treatment (such as oral corticosteroids, ciclosporin, azathioprine or methotrexate).

Phototherapy/PUVA was the most common therapeutic modality chosen as the first‐line option in the management of moderate‐to‐severe atopic eczema in adults (46%) (Table S2; see Supporting Information). Systemic therapy was chosen by 36% as the first‐line approach, but was the most commonly chosen second‐line approach (49%). A small number (5%) indicated they would never initiate or refer for phototherapy/PUVA, but all respondents indicated they would prescribe a systemic therapy as a first‐, second‐ or third‐line choice. Admission into hospital and referral for day‐case topical treatment were predominantly reserved for third‐line options.

The numbers of patients treated with the two most common therapeutic options are shown in Table S3 (se Supporting Information). Using these data and the formula above, the median numbers of patients treated per dermatologist over an average 3‐month period with phototherapy/PUVA and oral systemic therapy were calculated as 2·7 and 4·8, respectively.

### Phototherapy

**Figure 1:**
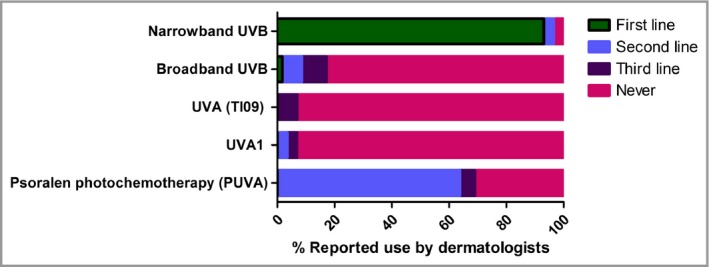
Relative reported priority for phototherapy selection by U.K. dermatologists in the management of moderate‐to‐severe atopic eczema in adults. UV, ultraviolet.

Figure 1 shows that of the 58 respondents using phototherapy/PUVA, the majority (93%) would choose narrowband UVB as the first‐line modality and PUVA as second line (64%). Interestingly, 83% of respondents would never consider broadband UVB and 93% would never consider UVA (TL09) or UVA1.

Respondents' own clinical experience (97% agreed or strongly agreed) and their knowledge of acute (90%) and chronic (95%) side‐effect profiles were major factors influencing choice of phototherapy for treatment (Table 1).

**Table 1:** Factors influencing consultant‐level dermatologists' choice of phototherapy/psoralen–ultraviolet A for moderate‐to‐severe atopic eczema in adults

	Strongly agree	Agree	Neutral	Disagree	Strongly disagree
Evidence base for treatment efficacy including support by randomized controlled trials	15 (26)	17 (29)	19 (33)	7 (12)	0
National or international guidelines	10 (17)	25 (43)	23 (40)	0	0
Expert opinion disseminated through lectures and clinical meetings	9 (16)	33 (57)	16 (28)	0	0
Own clinical experience	24 (41)	32 (55)	2 (3)	0	0
Acute side‐effect profile	18 (31)	34 (59)	5 (9)	1 (2)	0
Potential long‐term side‐effect profile	21 (36)	34 (59)	3 (5)	0	0
Patient choice	17 (29)	35 (60)	4 (7)	2 (3)	0

All values are *n* (%).

### Systemic treatments

**Figure 2:**
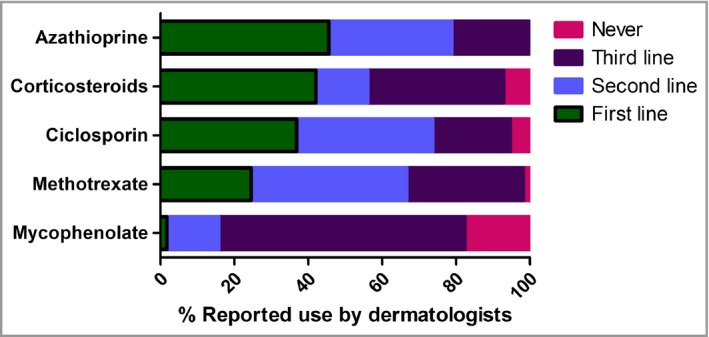
Relative reported priority of systemic therapies in the management of moderate‐to‐severe atopic eczema in adults.

As shown in Figure 2, azathioprine was the preferred first‐line systemic agent reported, ahead of oral corticosteroids, ciclosporin and methotrexate. Interestingly methotrexate was the most commonly used second‐line agent (Fig. 2). Although mycophenolate mofetil was rarely used as the first‐line agent, it was the preferred third‐line agent (Fig. 2). Very occasional use of systemic tetracyclines, doxycycline, high‐dose intravenous immunoglobulin, hydroxycarbamide, alitretinoin (if hands are involved) and thioguanine was also reported. The median numbers of patients started on systemic therapies per dermatologist over a typical 3‐month period were azathioprine 1·68, ciclosporin 1·53, methotrexate 1·36, mycophenolate mofetil 0·59 and oral corticosteroids 1·06 (calculated from the formula above and the data in Table S4; see Supporting Information).

**Figure 3:**
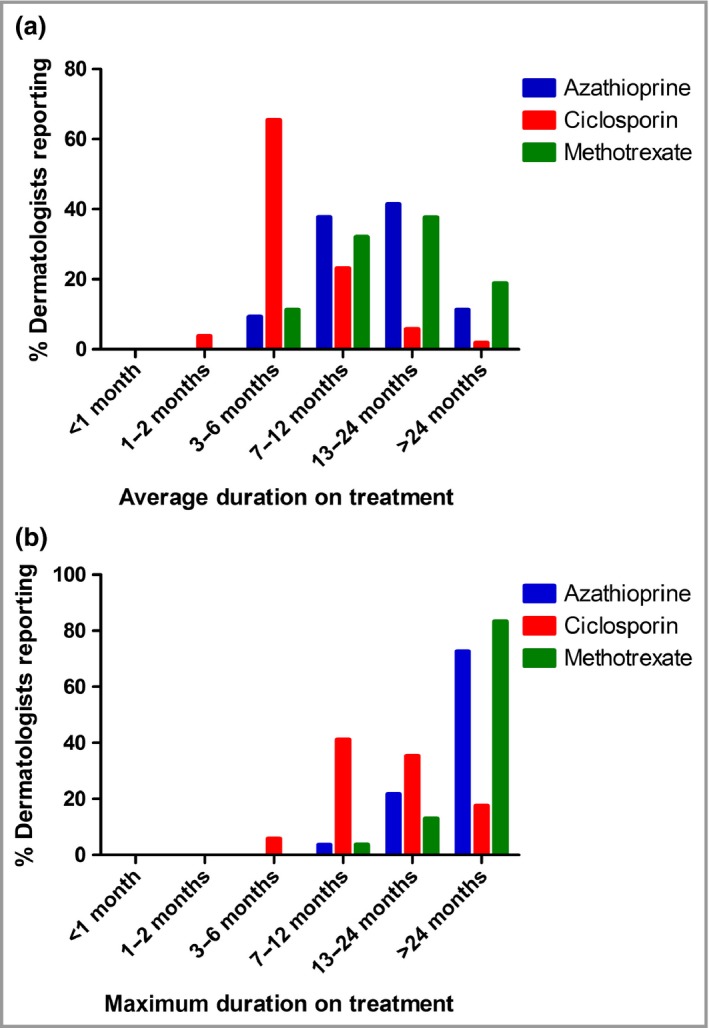
(a) Average and (b) maximum duration of treatment reported by U.K. dermatologists for specified therapeutic agents.

Marked differences in the average duration of treatment between agents were reported (Fig. 3a). On average, azathioprine treatment was continued for 13·8 months, compared with 5·8 months for ciclosporin. Methotrexate was continued for 15·1 months on average, similarly to azathioprine. Notably, the majority of respondents reported using ciclosporin for a maximum of 7–12 months, compared with > 24 months for azathioprine and methotrexate (Fig. 3b).

The main factors influencing dermatologists' choice of systemic agent for patients with moderate‐to‐severe atopic eczema are shown in Table 2. The most frequently reported factors were results of baseline tests and comorbidities, and respondents' own clinical experience of systemic agents. Dermatologists' knowledge of treatment efficacy and potential side‐effect profiles also featured prominently.

**Table 2:** Factors influencing dermatologists' choice of systemic therapy for moderate‐to‐severe atopic eczema in adults

	Strongly agree	Agree	Neutral	Disagree	Strongly disagree
Cost	5 (9)	6 (11)	24 (43)	12 (21)	9 (16)
Results of baseline tests and comorbidities	39 (70)	17 (30)	0	0	0
Medium‐term (> 3 months) efficacy and potential for maintenance treatment	29 (52)	26 (46)	1 (2)	0	0
Short‐term (< 3 months) efficacy	23 (41)	31 (55)	1 (2)	1 (2)	0
Acute side‐effect profile	26 (46)	27 (48)	2 (4)	0	1 (2)
Potential long‐term side‐effect profile	29 (52)	25 (45)	1 (2)	1 (2)	0
Evidence base for treatment efficacy including support by randomized controlled trials	13 (23)	29 (52)	13 (23)	1 (2)	0
National or international guidelines	11 (20)	30 (54)	14 (25)	1 (2)	0
Expert opinion disseminated through lectures and clinical meetings	12 (21)	36 (64)	7 (13)	1 (2)	0
Own clinical experience of systemic agents	31 (55)	25 (45)	0	0	0
Patient choice	25 (45)	26 (46)	4 (7)	1 (2)	0

All values are *n* (%).

When asked about factors influencing their choice of initial dose of oral systemic agent, 95% of the respondents either agreed or strongly agreed that thiopurine methyltransferase status at baseline would influence their choice.

### Unmet needs and future studies

Respondents were asked, ‘what is the most important currently unanswered question about the treatment of moderate‐to‐severe atopic eczema in adults?’ Three common themes emerged from the 47 free‐text responses. These can be summarized as, ‘which treatments are the most effective?’ (*n* = 24, 51%), ‘what alternatives are there to current approaches?’ (*n* = 15, 32%) and ‘how safe is long‐term maintenance treatment?’ (*n* = 10, 21%).

When questioned on the most important randomized controlled trial (RCT) that should be performed on treatment of moderate‐to‐severe atopic eczema in adults, the majority of the 45 respondents (*n* = 31, 69%) identified comparative studies between either two systemic treatments, or a systemic treatment and phototherapy. Respondents were then asked to describe, in their opinion, the most important RCT that should be performed in relation to systemic therapy, and 27 of 37 (73%) identified a trial involving methotrexate as the most important. Within this group, 18 respondents (49%) proposed a trial in which methotrexate would be compared with azathioprine, and six respondents (16%) proposed methotrexate vs. ciclosporin.

When questioned on the most important RCT that should be performed in relation to phototherapy, the greatest response (18 of 39 respondents, 46%) was for active‐comparative studies. Eight respondents (21%) proposed comparisons of one phototherapy with another, and seven of these (18% of the total) wished to compare TL01 UVA with PUVA. Another 10 (26%) suggested a comparison of any form of phototherapy with another treatment such as systemic drugs or topical steroids. Issues around duration of remission (*n* = 7, 18%) and dosimetry regimens (*n* = 4, 10%) were also raised.

## Discussion

Although atopic eczema is one of the most common dermatoses affecting adults that is treated by dermatologists,^14^, ^15^ the treatment modalities used, the hierarchy of their use in practice, and the rationale guiding therapeutic decision making are poorly understood. We found that U.K. dermatologists use phototherapy and systemic drugs to treat adults with recalcitrant moderate‐to‐severe atopic eczema, and that phototherapy/PUVA was the most common first‐line modality chosen. In line with trial evidence and published guidelines, narrowband UVB was the most common form of phototherapy used. Fewer dermatologists chose systemic therapy as a first‐line approach, but it was the most common modality used second line. Azathioprine was the most frequently prescribed systemic drug, even though ciclosporin is the only drug licensed for this indication. Azathioprine is used for maintenance therapy over longer periods than ciclosporin. Methotrexate, the most frequently used second‐line systemic, was also used for longer‐term maintenance therapy.

To the best of our knowledge, this is the first national survey to address the hierarchy of current treatment approaches by consultant‐level dermatologists for adults with refractory moderate‐to‐severe eczema. We received responses from a good cross‐section of consultant‐level dermatologists working in a variety of clinical situations and in different geographical locations across the U.K. One of the main shortcomings of this study is the low survey response rate. Although this is typical for online surveys,^16^, ^17^ and the relationship between response rate and nonresponse bias is not straightforward,^18^ this might have resulted in bias. As we selected our participants from the membership of the U.K. TREND and U.K. DCTN groups, we also selected a sample with a strong interest in research. This, combined with the low response rate, potentially limits the statistical generalizability of our findings. On the other hand, in areas of overlap with previously published surveys, our data appear consistent and aligned.^2^, ^19^, ^20^

Our finding that > 90% of the responding U.K. dermatologists would choose narrowband UVB as their first‐line phototherapy modality is in line with evidence from RCTs^4^ and recently published European guidelines.^21^ However, it is interesting that respondents’ own clinical experience and knowledge of acute and chronic side‐effect profiles (rather than their knowledge of the evidence base for treatment efficacy) appeared to be the major factors influencing their choice.

Among paediatric dermatologists and paediatricians in Europe,^2^ ciclosporin was the most commonly used first‐ and second‐line medication. However, notably there was clear regional variation, and within the U.K. azathioprine appeared to be the most prescribed first‐line systemic agent for children, although ciclosporin was almost as commonly used.^2^ Use of azathioprine as the first‐line choice of systemic is in line with a growing body of literature, including guidelines on the safe and effective prescription of azathioprine for dermatological conditions published by the BAD in 2011, and later studies.^5^, ^22^, ^23^, ^24^ Systematic reviews and European guidelines have also recommended consideration of azathioprine for treatment of refractory moderate‐to‐severe atopic eczema.^9^, ^25^ On the other hand, although treatment guidelines report a largely unfavourable risk‐to‐benefit ratio for the use of systemic corticosteroids,^9^ a surprisingly high percentage of respondents (42%) reported using systemic corticosteroids as the first‐line choice. This might reflect short‐term treatment, particularly if admission to hospital is not available, although participants were asked to consider their answers outwith the treatment of acute flares, and we did not ask about the duration of use of systemic steroids.

The reported average and maximum durations of therapy for azathioprine and ciclosporin provide insight into the different treatment approaches offered by these drugs. The reported maximum duration of use of ciclosporin is in line with the National Institute of Health and Care Excellence guidelines for psoriasis, which suggest a maximum of 1 year due to the risk of hypertension, renal impairment and cancer.^6^, ^7^, ^8^, ^11^ The average length of azathioprine treatment was considerably longer at 13·8 months, compared with 5·8 months for ciclosporin. Furthermore, the reported maximum treatment times with azathioprine indicate that the majority of our respondents considered it as a longer‐term (> 2‐year) treatment or maintenance option. Nevertheless, the BAD guidelines caution about the prolonged use of azathioprine in part because of uncertainty over cancer risk.^22^ Reassuringly, 95% of respondents were aware that thiopurine methyltransferase status should be checked prior to initiating azathioprine therapy.^22^ The factors reported as influencing respondents’ choice of systemic agent provide evidence that consultant dermatologists are aware of the risks and benefits involved with specific treatment modalities. Although the evidence base to support the efficacy of methotrexate^9^, ^24^, ^26^ in refractory moderate‐to‐severe atopic eczema is less robust than for azathioprine and ciclosporin,^9^ it appears at least to be equally as effective as azathioprine.^24^

The most important unanswered questions and research trial priorities identified by our survey of consultant dermatologists were consistent with a recent study specifically designed to identify and prioritize important research questions among patients, carers, clinicians and researchers for the treatment of atopic eczema.^19^ Our findings also align with a recently published eDelphi exercise performed by U.K. TREND that highlighted identification of novel systemic treatments for adult eczema as one of the top 10 translational research questions in inflammatory skin disease.^20^

We sought the views of our respondents on RCTs they would like to see conducted. Comparative studies between two systemic treatments or between a systemic treatment and phototherapy were identified as the most important RCTs that should be performed, followed by single‐agent placebo‐controlled studies. Methotrexate was the most commonly identified systemic for future trials. Another approach, particularly suited to addressing questions of long‐term safety and disease control, is to study large national patient cohorts, with long‐term follow‐up. Such a study is currently being set up under the auspices of U.K. TREND and will provide important comparative data not only on conventional systemics and phototherapy, but also on biological agents, once these have entered routine clinical practice.

## Supporting information


**Table S1.** Number of newly referred adults with moderate‐to‐severe atopic eczema seen personally by U.K. dermatologists over an average 3‐month period.
**Table S2.** Priorities of therapeutic options in the management of adult moderate‐to‐severe atopic eczema.
**Table S3.** Numbers of adults with moderate‐to‐severe atopic eczema initiated or referred for phototherapy/psoralen–ultraviolet A or oral systemic treatment over an average 3‐month period.
**Table S4.** Number of patients started on specific systemic treatments, per dermatologist, over an average 3‐month period.

**Appendix S1.** List of the full questions used in the survey.
