# Supplementary material for: Treatment of moderate‐to‐severe atopic eczema in adults within the U.K.: results of a national survey of dermatologists
Source: Br J Dermatol. 2017 Apr 16;176(6):1617–23. doi: 10.1111/bjd.15235 (PMC5516126; doi:10.1111/bjd.15235)
Supplement: Supplementary file 1 — Table S1. Number of newly referred adults with moderate‐to‐severe atopic eczema seen personally by U.K. dermatologists over an average 3‐month period. Table S2. Priorities of therapeutic options in the management of adult moderate‐to‐severe atopic eczema. Table S3. Numbers of adults with moderate‐to‐severe atopic eczema initiated or referred for phototherapy/psoralen–ultraviolet A or oral systemic treatment over an average 3‐month period. Table S4. Number of patients started on specific systemic treatments, per dermatologist, over an average 3‐month period. [file BJD-176-1617-s001.docx]

| Number of patients seen per three-month period | Number (%) of dermatologists reporting |
| --- | --- |
| <1 | 3 (4.9%) |
| 1-5 | 18 (29.5%) |
| 6-10 | 18 (29.5%) |
| 11-15 | 12 (19.7%) |
| >15 | 10 (16.4%) |

Table 1. Number of newly referred adults with moderate-to-severe atopic eczema seen personally by UK dermatologists over an average three-month period.

|  | Therapy selection, n (%) | | | |
| --- | --- | --- | --- | --- |
|  | Initiate or refer for phototherapy/PUVA | Prescribe systemic therapy | Refer for day case topical treatment | Admit into hospital |
| First-line | 28 (45.9) | 22 (36.1) | 13 (21.3) | 2 (3.3) |
| Second-line | 23 (37.7) | 30 (49.2) | 7 (11.5) | 6 (9.8) |
| Third-line | 7 (11.5) | 9 (14.8) | 20 (32.8) | 33 (54.1) |
| Never | 3 (4.9) | 0 (0) | 21 (34.4) | 20 (32.8) |

Table 2. Priorities of therapeutic options in the management of adult moderate-to-severe atopic eczema.

|  | Number (%) of dermatologists reporting | |
| --- | --- | --- |
| Number of patients per three-month period | Phototherapy/PUVA | Systemic therapy |
| <1 | 18 (31.0) | 4 (7.0) |
| 1-2 | 13 (22.4) | 13 (22.8) |
| 3-5 | 16 (27.6) | 26 (45.6) |
| 6-10 | 9 (15.5) | 11 (19.3) |
| >10 | 2 (3.4) | 3 (5.3) |
| Total | 58 | 57 |

Table 3. Numbers of adults with moderate-to-severe atopic eczema initiated or referred for phototherapy/PUVA or oral systemic treatment over an average three-month period.

| Reported use of systemic therapies, n (%) | | | | | |
| --- | --- | --- | --- | --- | --- |
| Number of new patients per three- month period | Azathioprine | Ciclosporin | Methotrexate | Mycophenolate mofetil | Oral corticosteroids |
| <1 | 18 (32.7%) | 21 (40.4%) | 23 (42.6%) | 36 (83.7%) | 24 (48.0%) |
| 1-2 | 28 (50.9%) | 19 (36.5%) | 22 (40.7%) | 7 (16.3%) | 17 (34.0%) |
| 3-5 | 7 (12.7%) | 11 (21.2%) | 8 (14.8%) | 0 (0.0%) | 7 (14.0%) |
| 6-10 | 2 (3.6%) | 1 (1.9%) | 1 (1.9%) | 0 (0.0%) | 2 (4.0%) |
| >10 | 0 (0.0%) | 0 (0.0%) | 0 (0.0%) | 0 (0.0%) | 0 (0.0%) |

Table 4. Number of patients started on specific systemic treatments, per dermatologist, over an average three-month period.
