# Supplementary material for: Treatment of moderate‐to‐severe atopic eczema in adults within the U.K.: results of a national survey of dermatologists
Source: Br J Dermatol. 2017 Apr 16;176(6):1617–23. doi: 10.1111/bjd.15235 (PMC5516126; doi:10.1111/bjd.15235)
Supplement: Supplementary file 2 — Appendix S1. List of the full questions used in the survey. [file BJD-176-1617-s002.pdf]

The treatment of moderate-severe atopic eczema in adults

Thank you for agreeing to participate in this survey which aims to collect data on current therapeutic approaches for adult patients with moderate-to-severe atopic eczema and factors that influence choice of therapy.

This questionnaire is being conducted by Newcastle University in collaboration with UK TREND (the UK Translational Research Network in Dermatology) and UK DCTN (UK Dermatology Clinical Trials Network). It is independent from the pharmaceutical industry.

The survey has 5 sections and will take approximately 15 minutes to complete in total. We do not ask for identifying information and results will be reported anonymously. The information collected will help us design studies to improve the treatment of atopic eczema.

Definitions

For the purpose of this survey, we define "moderate-to-severe atopic eczema" as that which is not adequately controlled by standard and optimised topical treatment (including emollients, topical steroids and topical calcineurin inhibitors), apart from occasional short-term flares caused by skin infection for example.

Section 1: Demographics

1. Please indicate your current position
- o Consultant dermatologist
- o Clinical academic (honorary consultant dermatologist)
- o Associate specialist in dermatology
- o Other (please specify below)

Other…………………………………..

2. In which country did you receive the majority of your specialty training?

- o United Kingdom
- o France
- o Germany
- o Ireland
- o Italy
- o Spain
- o Sweden
- o The Netherlands
- o Other (please specify below)

Other………………………………...

3. From which dermatology organisation/network did you receive the invitation to the questionnaire?
- o UK TREND
- o UK DCTN

4. In which city do you currently work? …………………………………………………………………

5. Where do you see the majority of your adult dermatology patients?

- o University teaching hospital
- o General hospital
- o Private practice
- o Community
- o Other (please specify below)

Other…………………………………..

6. Please rank the ethnic mix of your adult dermatology patients. If two ethnic groups are very similar, please consider which group will be slightly larger over a year.

	First (Majority)	Second (Frequently)	Third (Occasionally)	Fourth (Rarely)	Fifth (Very rarely)
White					
Asian (e.g. Indian)					
Black African					
Black Caribbean					
Chinese or other East Asian					

7. Please estimate how many newly referred adults with moderate-to-severe atopic eczema, as defined earlier, you personally see over an average 3-month period.

- o <1 patient
- o 1-5 patients
- o 6-10 patients
- o 11-15 patients
- o >15 patients

8. In adult patients with moderate-to-severe atopic eczema do you ever use/refer for phototherapy/PUVA or initiate oral systemic treatment (i.e. oral corticosteroids, ciclosporin, azathioprine, methotrexate)?

- o Yes
- o No

Section 2: Treatment options

Considering moderate-to-severe atopic eczema (ie not adequately controlled by standard and optimised topical treatments), please answer the following questions about your treatment of choice.

9. Please indicate the first, second and third line therapeutic options that you would use in the management of an adult with moderate-to-severe atopic eczema. Note that this is outwith the context of an acute flare.

	First line	Second line	Third line	Never
Admit into hospital				
Initiate or refer for phototherapy/PUVA				
Prescribe systemic therapy				
Refer for day case topical treatment				

Section 3: Phototherapy

Considering moderate-to-severe atopic eczema (ie not adequately controlled by standard and optimised topical treatments) as defined earlier, please answer the following questions about your treatment of choice.

10. Please indicate your first, second and third line therapeutic options in adult patients with moderate-to-severe atopic eczema being considered for phototherapy/PUVA.

	First line	Second line	Third line	Never
Broad-band UVB				
Narrow-band UVB				
Psoralen photochemotherapy (PUVA)				
UVA (Tl09)				
UVA1				

11. Please estimate how many adults with moderate-to-severe atopic eczema you personally treat with or refer for phototherapy/PUVA over an average 3-month period.

- o <1 patient
- o 1-2 patients
- o 3-5 patients

- 6-10 patients
- >10 patients

12. How many adult patients with moderate-to-severe atopic eczema would be treated in your DEPARTMENT with phototherapy per annum?

- <1
- 1-5
- 6-20
- 21-50
- 51-100
- >100
- Don't know

13. In general, the main factors that influence your choice of phototherapy for treatment of moderate-to-severe atopic eczema referred for phototherapy/PUVA would be best described as:

	Strongly agree	Agree	Neutral	Disagree	Strongly disagree
My knowledge of the evidence base for treatment efficacy including support by RCTs					
My knowledge of national/international guidelines					
My knowledge of expert opinion disseminated through lectures/clinical meetings					
My own clinical experience					
My knowledge of acute side effect profile					
My knowledge of potential long-term side effect profile					
Patient choice					

Section 4: Systemic treatments

Considering moderate-to-severe atopic eczema (ie not adequately controlled by standard and optimised topical treatments), please answer the following questions about your treatment of choice.

14. Do you personally ever initiate oral systemic treatment (i.e. azathioprine, ciclosporin, methotrexate, mycophenolate mofetil, oral corticosteroids) in adults with moderate-to-severe eczema?
- o Yes
- o No

Section 4: Systemic treatments

15. Please estimate how many adults with moderate-to-severe atopic eczema you personally treat with or refer for oral systemic treatment over an average 3-month period.
- o <1 patient
- o 1-2 patients
- o 3-5 patients
- o 6-10 patients
- o >10 patients

16. How many adult patients with moderate-to-severe atopic eczema would be treated in your DEPARTMENT with oral systemic treatment per annum?
- o <1
- o 1-5
- o 6-20
- o 21-50
- o 51-100
- o >100
- o Don’t know

17. Please indicate your first, second and third line therapeutic options in adult patients with moderate-to-severe atopic eczema being considered for systemic therapy.

	First line	Second line	Third line	Never
Azathioprine				
Ciclosporin				
Methotrexate				
Mycophenolate mofetil				
Oral corticosteroids				
Other				

Section 4a: Influence on choice

18. In general, the main factors that influence your choice of systemic agent in patients with moderate-to-severe atopic eczema being considered for systemic therapy are best described as:

	Strongly agree	Agree	Neutral	Disagree	Strongly disagree
Cost					
Results of baseline tests and co-morbidities					
My knowledge of medium term (>3 months) efficacy and potential for maintenance treatment					
My knowledge of short term (<3 months) efficacy					
My knowledge of acute side effect profile					
My knowledge of potential long-term side effect profile					
My knowledge of the evidence base for treatment efficacy including support by RCTs					
My knowledge of national/international guidelines					
My knowledge of expert opinion disseminated through lectures/clinical meetings					
My own clinical experience of systemic agents					
Patient choice					

Section 4b: Azathioprine

If you indicated that you would use azathioprine in the management of moderate-to-severe atopic eczema as defined earlier, please answer the following questions.

If you do not use Azathioprine please scroll down and press next.

19. To what extent would you agree that the following statements are the main factors that influence your choice of initial dose?

	Strongly agree	Agree	Neutral	Disagree	Strongly disagree
TPMT level at baseline					
Patient weight at baseline					
Other					

20. To what extent would you agree that the following statements are the main factors that influence your choice of maximum dose?

	Strongly agree	Agree	Neutral	Disagree	Strongly disagree
Reported side effects					
Abnormal laboratory tests					
Measurement of active metabolites (TGNs) in blood					
Other					

21. What would you anticipate as being the AVERAGE and MAXIMUM duration of treatment on azathioprine?

	<1 month	1-2 months	3-6 months	7-12 months	13-24 months	>24 months
Average duration						
Maximum duration						

22. Please estimate how many adults with moderate-to-severe atopic eczema not adequately controlled by standard and optimised topical treatment, you personally start on azathioprine during an average 3-month period

- o <1 patient
- o 1-2 patients
- o 3-5 patients
- o 6-10 patients

- >10 patients

Section 4c: Ciclosporin

If you indicated that you would use ciclosporin in the management of moderate-to-severe atopic eczema as defined earlier, please answer the following questions.

If you do not use ciclosporin please scroll down and press next.

23. Please indicate the INITIAL dose and MAXIMUM dose of ciclosporin that you would generally use.

	<2 mg/kg/day in divided doses	2-3 mg/kg/day in divided doses	3-5 mg/kg/day in divided doses	>5 mg/kg/day in divided doses
Initial dose				
Maximum dose				

24. What would you anticipate as being the AVERAGE and MAXIMUM duration of treatment on ciclosporin?

	<1 month	1-2 months	3-6 months	7-12 months	13-24 months	>24 months
Average duration						
Maximum duration						

25. Please estimate how many adults with moderate-to-severe atopic eczema you personally start on ciclosporin during an average 3-month period.

- <1 patient
- 1-2 patients
- 3-5 patients
- 6-10 patients
- >10 patients

Section 4d: Methotrexate

If you indicated that you would use methotrexate in the management of moderate-to-severe atopic eczema as defined earlier, please answer the following questions.

If you do not use methotrexate please scroll down and press next.

26. Please indicate the INITIAL dose and MAXIMUM dose of methotrexate that you would generally use.

	5 mg weekly	10 mg weekly	15 mg weekly	20 mg weekly	25 mg weekly	>25 mg weekly
Initial dose						
Maximum dose						

27. What would you anticipate as being the AVERAGE and MAXIMUM duration of treatment on methotrexate?

	<1 month	1-2 months	3-6 months	7-12 months	13-24 months	>24 months
Average duration						
Maximum duration						

28. Please estimate how many adults with moderate-to-severe atopic eczema as defined earlier, you personally start on methotrexate during an average 3-month period.

- <1 patient
- 1-2 patients
- 3-5 patients
- 6-10 patients
- >10 patients

Section 4e: Mycophenolate mofetil

If you indicated that you would use mycophenolate mofetil in the management of moderate-to-severe atopic eczema as defined earlier, please answer the following questions.

If you do not use mycophenolate mofetil please scroll down and press next.

29. Please indicate the INITIAL dose and MAXIMUM dose of mycophenolate mofetil that you would generally use.

	1g daily in divided doses	2g daily in divided doses	3g daily in divided doses	4g daily in divided doses	5g daily in divided doses
Initial dose					
Maximum dose					

30. What would you anticipate as being the AVERAGE and MAXIMUM duration of treatment on mycophenolate mofetil?

	<1 month	1-2 months	3-6 months	7-12 months	13-24 months	>24 months
Average duration						
Maximum duration						

31. Please estimate how many adults with moderate-to-severe atopic eczema you personally start on mycophenolate mofetil during an average 3-month period.
- o <1 patient
- o 1-2 patients
- o 3-5 patients
- o 6-10 patients
- o >10 patients

Section 4f: Oral corticosteroids

If you indicated that you would use oral corticosteroids in the management of moderate-to-severe atopic eczema as defined earlier, please answer the following questions.

If you do not use oral corticosteroids please scroll down and press next.

32. Please indicate the INITIAL dose and MAXIMUM dose that you would generally use.

	<5 mg daily	5-9 mg daily	10-14 mg daily	15-19 mg daily	20-24 mg daily	≥25 mg daily
Initial dose						
Maximum dose						

33. Please indicate the AVERAGE and MAXIMUM duration of treatment and regimen used to discontinue treatment.

	<1 month	1-2 months	3-6 months	7-12 months	13-24 months	>24 months
Average duration						
Maximum duration						

34. Please estimate how many adults with moderate-to-severe atopic eczema, you personally start on oral corticosteroids during an average 3-month period

- <1 patient
- 1-2 patients
- 3-5 patients
- 6-10 patients
- >10 patients

Section 4g: Other

If you indicated that you would use "other" in the management of moderate-to-severe atopic eczema as defined earlier, please answer the following questions.

If you do not use other treatments please scroll down and press next.

35. Please list the "other" drug or drugs.

……………………………………………….

36. Please indicate the INITIAL dose and MAXIMUM dose of treatment.

Initial dose…………………………………

Maximum dose………………………………………..

37. Please indicate the AVERAGE and MAXIMUM duration of treatment and regimen used to discontinue treatment.

	<1 month	1-2 months	3-6 months	7-12 months	13-24 months	>24 months
Average duration						
Maximum duration						

38. Please estimate how many adults with moderate-to-severe atopic eczema, you personally start on "other" during an average 3-month period

- <1 patient
- 1-2 patients
- 3-5 patients
- 6-10 patients
- >10 patients

Section 5: Future work

And finally, thinking about the future …

39. In your opinion, what is the most important currently unanswered question about the treatment of moderate-to-severe atopic eczema in adults?

………………………………………..

40. In your opinion, what is the most important RCT that should be performed in moderate-to-severe atopic eczema in adults?

…………………………………………………………

41. In your opinion, what is the most important RCT that should be performed in relation to phototherapy for moderate-to-severe atopic eczema in adults?

……………………………………………………………….

42. In your opinion, what is the most important RCT that should be performed in relation to systemic therapy of moderate-to-severe atopic eczema in adults?

……………………………………………………………..

43. If you would be interested in becoming a recruiting centre for future trials in atopic eczema please enter your name and email address in the box below.

……………………………………………………………..

44. Please add any further comments or suggestions in the box below.

……………………………………………………………..

45. If you would be happy to be contacted to discuss any of the issues raised in this survey please add your preferred contact details in the box below. Please note that the results of the survey will not be linked to individuals or email accounts.

………………………………………………………………..

Thank you for completing the survey.
